# Brachial plexus birth injury and cerebral palsy lead to a common contracture phenotype characterized by reduced functional muscle length and strength

**DOI:** 10.3389/fresc.2022.983159

**Published:** 2022-08-16

**Authors:** Sia Nikolaou, Micah C. Garcia, Jason T. Long, Allison J. Allgier, Qingnian Goh, Roger Cornwall

**Affiliations:** ^1^ Cornwall/Goh Lab, Division of Orthopaedic Surgery, Cincinnati Children’s Hospital Medical Center, Cincinnati, OH, United States; ^2^ Motion Analysis Lab, Division of Occupational Therapy and Physical Therapy, Cincinnati Children’s Hospital Medical Center, Cincinnati, OH, United States; ^3^ Department of Orthopedic Surgery, University of Cincinnati College of Medicine, Cincinnati, OH, United States; ^4^ Division of Developmental Biology, Cincinnati Children’s Hospital Medical Center, Cincinnati, OH, United States

**Keywords:** cerebral palsy, brachial plexus birth injury, contracture, isokinetic strength, sarcomere length, muscle length

## Abstract

**Introduction:**

Brachial plexus birth injury (BPBI) and cerebral palsy (CP) both cause disabling contractures for which no curative treatments exist, largely because contracture pathophysiology is incompletely understood. The distinct neurologic nature of BPBI and CP suggest different potential contracture etiologies, although imbalanced muscle strength and insufficient muscle length have been variably implicated. The current study directly compares the muscle phenotype of elbow flexion contractures in human subjects with BPBI and CP to test the hypothesis that both conditions cause contractures characterized by a deficit in muscle length rather than an excess in muscle strength.

**Methods:**

Subjects over 6 years of age with unilateral BPBI or hemiplegic CP, and with elbow flexion contractures greater than 10 degrees on the affected side, underwent bilateral elbow flexion isokinetic strength testing to identify peak torque and impulse, or area under the torque-angle curve. Subjects then underwent needle microendoscopic sarcomere length measurement of bilateral biceps brachii muscles at symmetric joint angles.

**Results:**

In five subjects with unilateral BPBI and five with hemiplegic CP, peak torque and impulse were significantly lower on the affected versus unaffected sides, with no differences between BPBI and CP subjects in the percent reduction of either strength measurement. In both BPBI and CP, the percent reduction of impulse was significantly greater than that of peak torque, consistent with functionally shorter muscles. Similarly, in both conditions, affected muscles had significantly longer sarcomeres than unaffected muscles at symmetric joint angles, indicating fewer sarcomeres in series, with no differences between BPBI and CP subjects in relative sarcomere overstretch.

**Discussion:**

The current study reveals a common phenotype of muscle contracture in BPBI and CP, with contractures in both conditions characterized by a similar deficit in muscle length rather than an excess in muscle strength. These findings support contracture treatments that lengthen rather than weaken affected muscles. Moreover, the discovery of a common contracture phenotype between CP and BPBI challenges the presumed dichotomy between upper and lower motor neuron lesions in contracture pathogenesis, instead revealing the broader concept of “myobrevopathy”, or disorder of short muscle, warranting increased investigation into the poorly understood mechanisms regulating muscle length.

## Introduction

Cerebral palsy and brachial plexus birth injury are the two most common causes of neonatal-onset neuromuscular dysfunction in childhood, affecting a combined 5 per 1,000 children (1, 2). Cerebral palsy (CP) refers to a broad group of neuromotor disorders wherein a perinatal brain insult leads to chronic motor dysfunction, including skeletal muscle weakness, spasticity, and/or impaired motor coordination (3). Brachial Plexus Birth Injury (BPBI) is a traumatic, delivery-related peripheral nerve injury involving the nerve roots of the brachial plexus, leading to flaccid paralysis of muscles in the upper limb, with neurologic dysfunction being permanent in 20%–40% of affected individuals (4). Despite differing in the type of neurologic lesion (upper vs. lower motor neuron; central vs. peripheral nervous system), both CP and BPBI lead to similar secondary muscle contractures that limit the passive range of motion of affected limbs (4–6). These contractures are a major source of disability and are frequent indications for rehabilitative and surgical intervention (4, 7–9). The contractures also progressively worsen throughout childhood and lead to progressive and incurable bone and joint deformity (10, 11). However, existing contracture treatments used in either condition cannot restore normal joint motion, and can risk further loss of function by further weakening the muscles responsible for the contractures (12–15). This lack of curative treatments for muscle contractures in CP and BPBI is complicated by the fact that the pathogenesis of contracture formation in neuromuscular conditions is unclear (16). On a fundamental level, it is unknown whether these contractures are the mechanical consequence of altered limb mobility or the biological consequence of altered muscle innervation during postnatal development.

Mechanical theories of contracture pathogenesis in CP and BPBI primarily involve abnormal joint posturing from imbalanced muscle strength (8, 17). In CP, this muscle imbalance results from spasticty, whereas in BPBI, strength imbalance results from unequal denervation across agonist/antagonist muscle group pairs. This muscle strength imbalance theory of contracture pathogenesis has led to the widespread use of muscle-weakening treatments such as chemodenervation to reduce the presumably excess strength of muscles responsible for contractures (18, 19). However, the muscle imbalance theory of contracture pathogenesis is insufficient to explain certain clinical scenarios in both conditions. For instance, in CP, interventions that eliminate muscle spasticity do not prevent or relieve contractures (20). Additionally, in BPBI, muscle imbalance cannot explain paradoxical contractures such as the elbow flexion contracture that develops in the setting of elbow flexor paralysis with preserved elbow extensor function (21).

Alternatively, biological theories of contracture pathogenesis involve impaired longitudinal growth of abnormally innervated muscles. In humans with CP (22, 23), muscles involved in contractures have been found to be functionally shorter than normal, with this functional shortening characterized by overstretched sarcomeres at controlled joint angles, suggesting too few sarcomeres in series. Similar findings have been seen in contractures in rodent models of BPBI (24, 25). Therefore it is conceiveable that contractures in both conditions are caused by a deficit in muscle length rather than an excess in muscle strength. However, muscle length has never been directly compared in contractures in CP and BPBI, as the findings in BPBI have been limited to animal models, with only slack sarcomere length being measured in humans with BPBI (26).

Directly comparing the muscle contracture phenotypes in terms of both strength and length in CP and BPBI could help to elucidate the relative contributions of mechanical and developmental factors to contracture pathogenesis. Are contractures in these two disparate neurological conditions fundamentally distinct or similar at the muscle level? Fundamental differences in the muscle phenotype between CP and BPBI would support a role for the different mechanical effects of spastic vs. flaccid paralysis. Conversely, identification of a final common phenotype of muscle contracture, independent of spastic vs. flaccid paralysis, would support a role for muscle growth impairment from disruption of normal neuromuscular development.

Thus the current study directly compares strength and functional muscle length in humans with elbow flexion contractures caused by BPBI and CP. We utilize isokinetic strength testing and minimally invasive *in vivo* sarcomere length measurement in subjects with unilateral BPBI and hemiplegic CP to compare contractured and contralateral non-contractured muscles within subjects and across conditions. With this approach, the study aims to test the hypothesis that both CP and BPBI lead to a common contracture phenotype of short, weak muscles. Elucidation of such a common phenotype could have important ramifications for the translation of discoveries and treatment approaches across these and potentially other seemingly disparate neuromuscular conditions.

## Materials and methods

### Subjects

Subjects at least 6 years of age with unilateral BPBI or spastic hemiplegic CP, and with an elbow flexion contracture on the involved side between 10° and 90° were included. A minimum age of 6 years was required for reliable isokinetic testing, and this age coincides with the onset and progression of elbow flexion contractures in both conditions (6, 21). Subjects meeting these criteria were recruited from interdisciplinary clinics if they were indicated for any surgical procedure under general anesthesia, even if unrelated to their elbow flexion contracture. Such surgical procedures afforded the opportunity for sarcomere length measurement under general anesthesia to eliminate the discomfort of needle microendoscopy and to allow control of elbow position without muscle contraction; the pre-existing surgical indication eliminated the need for study-specific general anesthesia. Patients with a history of surgical or recent (within 6 months) chemical (botulinum toxin) treatment of an elbow flexion contracture were excluded.

### Isokinetic strength testing

Upon study enrollment, elbow flexion/extension torque was measured bilaterally with surface electromyography to assess muscle activation and co-contraction. Trigno surface EMG electrodes (Delsys, Inc.; Natick, MA) were affixed over the biceps and triceps muscle bellies bilaterally. Isokinetic strength was collected using the Biodex System 4 (Biodex Medical Systems, Inc.; Shirley, NY) configured for elbow flexion/extension. The subject performed a series of 10 flexion/extension cycles at two speeds (60°/s and 120°/s). The subject was encouraged to perform the cycles as fast as possible. Testing was performed bilaterally, with the affected side tested first.

During each series of flexion/extension cycles, continuous angular position and torque data were collected from the Biodex System 4 (*f*_s _= 120 Hz). Synchronous muscle activity data was collected from each muscle of interest *via* the Delsys Trigno electrodes (*f*_s _= 800 Hz). Each time-series of flexion/extension cycles was subdivided into ten individual cycles, and the initial and terminal cycles were removed. The remaining 8 cycles were used for analysis. For each muscle within each cycle, elbow flexion angle/torque measures from the Biodex data (peak torque, time to peak torque, angle at peak torque, and area under the angle/torque curve, or impulse) were identified. Elbow extension strength was not analyzed as it was confounded by gravity, which could not be reliably eliminated using an abducted shoulder position in the Biodex apparatus given the subjects’ limited shoulder mobility.

EMG data were full-wave rectified and bandpass filtered (10–400 Hz) to assess amplitude characteristics throughout each cycle. Periods of muscle activity were identified and compared temporally to torque-angle data to characterize the presence of agonist/antagonist behavior during the flexion/extension cycle.

### Sarcomere length measurement

**Figure 1:**
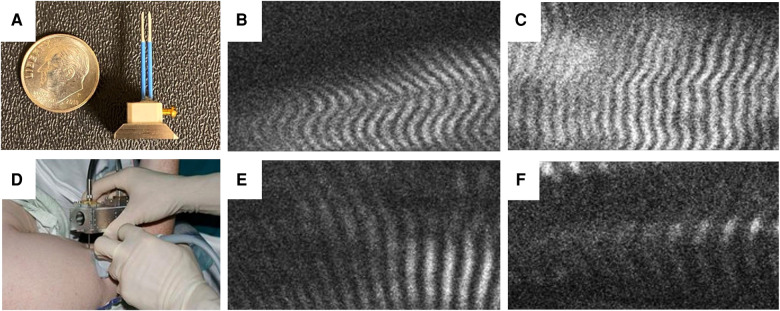
Needle microendoscopy of muscle sarcomeres. (**A**) Microendoscopic probe consisting of two 20-guage needles inserted into biceps muscle while mounted on handheld microscope (**B**) allows visualization of sarcomeres in real time as depicted from the unaffected and affected muscles in a subject with BPBI (**C,D**, respectively) and a subject with CP (**E,F**, respectively).

The true functional length of a muscle is defined by the total number of sarcomeres in series, which cannot be measured in a whole muscle. However, when two otherwise identical muscles of unequal functional length (different numbers of sarcomeres in series) are subjected to the same joint position, the muscle with the fewer sarcomeres in series will have to elongate its sarcomeres to a greater degree to accommodate that joint position. Thus, relative sarcomere overstretch is an indicator of functional muscle shortening (27). Thus, within 4 weeks following isokinetic strength testing, subjects underwent elbow passive range of motion and sarcomere length measurement while under general anesthesia, with paralytic agents used for anesthesia induction eliminating muscle contraction as a confounding variable. Elbow passive range of motion was measured bilaterally with protractor goniometry with the limbs positioned in shoulder adduction and neutral shoulder and forearm rotation. The elbows were then symmetrically positioned at the maximum extension angle of the affected side. Biceps brachii average sarcomere lengths were measured using needle microendoscopy. A needle micro-endoscope probe (Zebrascope, Zebra Medical Technologies, Mountain View, California) (28) was inserted into bilateral biceps brachii muscles, recording images of sarcomeres from multiple fibers per muscle (Figure 1). On the resulting images, six distinct muscle fibers were identified per muscle. For each fiber, sarcomere lengths were eassessed by measuring the total length of 5 consecutive sarcomeres and dividing by 5. The resulting sarcomere lengths for the six sampled fibers were averaged to obtain each muscle's average sarcomere length.

### Statistical analysis

Continuous variables were compared using paired t-tests for side-to-side comparisons and unpaired t-tests for between subject comparisons. Data are presented as as mean ± SD. The degree of significance between data sets is depicted as follows: *p < 0.05, **p < 0.01, ***p < 0.001, and ****p < 0.0001. Variance data from previous work (24, 25, 29) and other published data (22, 30, 31) were used to calculate sample sizes to achieve at least 80% power to detect a sarcomere length difference of at least 0.5 µm between the involved and control limb. All statistical tests were performed with Prism 8 software (GraphPad).

## Results

### Subjects

**Figure 2:**
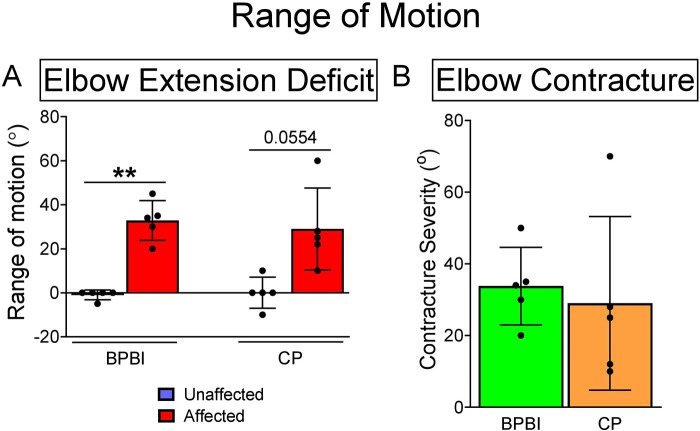
Elbow range of motion in BPBI and CP subjects. (**A**) Maximum passive elbow extension deficit in degrees for both BPBI and CP subjects, where full extension equals 0 degrees and hyperextension is negative. All subjects lacked at least 10 degrees from full extension on the affected sides. (**B**) Elbow flexion contracture severity, defined as the difference in maximum passive extension between affected and unaffected sides. All subjects had at least a 10 degree flexion contracture on the affected side. **p < 0.01.

Five subjects with spastic hemiplegic CP (2 males, 3 females, mean age 15.2 years, age range 14–16 years) and five subjects with unilateral BPBI (4 males, 1 female, mean age 13.8 years, age range 7–19 years) were included in the study. Etiologies of CP included perinatal/intrauterine stroke in 3 subjects, schizencephaly with intracranial hemorrhage in 1, and idiopathic in 1. In all CP subjects, the neurologic onset was perinatal or antenatal, as opposed to later anoxic, traumatic, or surgical (e.g. hemispherectomy for seizures) etiologies. By definition, all cases of BPBI were birth-related rather than later traumatic injuries. Six prior subjects were excluded due to the need for optimization of the microendoscopy technology and technique, given that this study used the first commercially available Zebrascope as well as custom microendoscopy probes. As the inclusion criteria required at least a 10-degree elbow flexion contracture, all subjects in both groups had at least a 10-degree loss of maximum passive elbow extension on the affected side versus the control side (Figure 2A). No subjects in either group had a loss of passive elbow flexion on the affected side compared to the contralateral side. Elbow flexion contracture severity, defined as the side-to-side difference in maximum passive elbow extension, averaged 34° (range 20–50°) in the BPBI subjects, and 29° (range 10–70°) in the CP subjects (Figure 2B).

### Isokinetic strength testing

**Figure 3:**
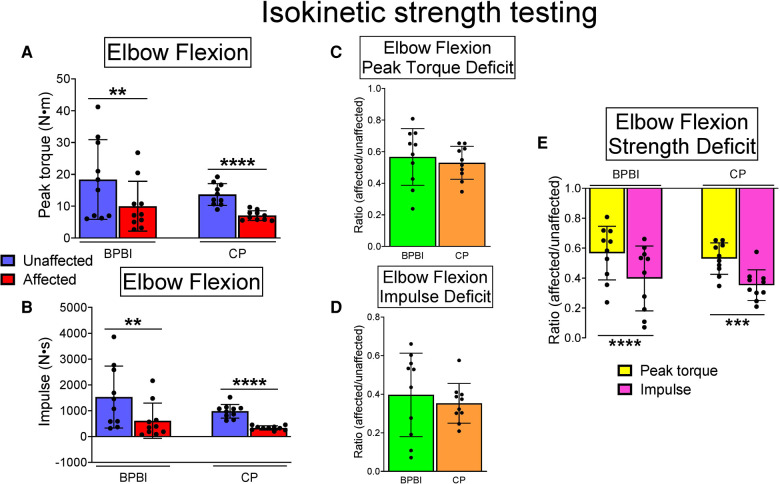
Isokinetic elbow flexion strength testing in BPBI and CP subjects, with 60 m/s and 120 m/s data pooled. (**A**) Peak torque was lower on affected versus unaffected sides for both BPBI and CP subjects. (**B**) Impulse, or area under the torque-angle curve, also known as total work, was reduced on the affected versus unaffected sides for both BPBI and CP subjects. The deficit in peak torque (**C**) and impulse (**D**) did not differ between BPBI and CP subjects. (**E**) The deficit in impulse was greater than the deficit in peak torque for both BPBI and CP subjects. **p < 0.01, ***p < 0.001, ****p < 0.0001.

In both conditions, affected limbs had lower elbow flexion peak torque than unaffected limbs (BPBI: 9.98 vs. 18.36 Nm, respectively, *p* < 0.01; CP: 7.05 vs. 12.94 Nm, respectively, *p* < 0.0001, Figure 3A). Affected limbs also had reduced impulse, or area under the torque-angle curve, than unaffected limbs (BPBI: 609.59 vs. 1527.63 Ns, respectively, *p* < 0.01; CP: 331.49 vs. 979.10 Ns, respectively, *p* < 0.0001, Figure 3B). There were no differences between BPBI and CP subjects in the percent reduction in peak torque (43.34 vs. 47.01%, respectively, *p* = 0.58, Figure 3C) or percent reduction in impulse (60.34 vs. 64.73%, respectively, *p* = 0.57, Figure 3D). For both CP and BPBI subjects, the percent reduction in impulse was greater than the percent reduction in peak torque (BPBI: 60.34 vs. 43.34%, respectively, *p* < 0.0001; CP: 64.73 vs. 47.01%, respectively, *p* < 0.001, Figure 3E). This finding indicates that affected limbs had not only lower maximum strength but also narrower dispersion of torque across their arc of motion, consistent with functionally shorter muscles.

In 3 BPBI subjects and 2 CP subjects, biceps and triceps muscles displayed reciprocal contraction on surface EMG during flexion-extension cycles, whereas 2 BPBI and 2 CP subjects demonstrated co-contraction. Usable EMG data were not available for one CP subject. However, the subjects with co-contraction had neither the lowest peak torques nor the lowest impulse among their respective groups, arguing against co-contraction as a sole cause of reduced elbow flexion torque overall.

### Sarcomere length measurement

**Figure 4:**
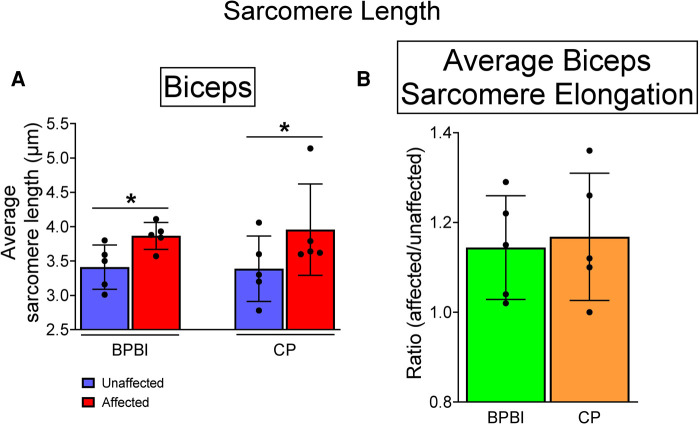
Sarcomere lengths in BPBI and CP subjects. (**A**) Average sarcomere lengths for biceps brachii muscles were longer on the affected versus unaffected sides for both BPBI and CP subjects. (**B**) The degree of sarcomere elongation, or overstretch, did not differ between BPBI and CP subjects. *p < 0.05.

In both BPBI and CP subjects, average sarcomere lengths were significantly longer in the affected versus unaffected biceps at symmetric joint angles (BPBI: 3.87 vs. 3.47 µm, respectively, *p* < 0.05; CP: 3.96 vs. 3.39 µm, respectively, *p* < 0.05, Figure 4A). This sarcomere elongation indicates overstretched (and thus functionally shorter) biceps muscles in the affected limb. The average percent increase in sarcomere length on the affected side did not differ between BPBI and CP (14.4% versus 16.8%, respectively, *p* = 0.78; Figure 4B).

## Discussion

The current study directly compares the contracture phenotype in BPBI and CP, demonstrating that contractures in both conditions are characterized by a remarkably similar reduction in both muscle length and strength. This discovery of a common phenotype bears several implications for the understanding of and approach to contractures in both BPBI and CP, and possibly other neuromuscular conditions.

First the similarly reduced muscle length in BPBI and CP contractures supports a final common pathway of longitudinal muscle growth impairment following even disparate perinatal neurologic insults. Impairment in longitudinal growth could also explain the similarly progressive nature of contractures throughout childhood in both CP (6, 9, 32, 33) and BPBI (21, 34). Impaired growth has been previously identified and postulated as causative of contractures in both CP and BPBI, but in different models and using different techniques. In humans subjects with CP, biopsies of various muscles responsible for contractures reveal greater sarcomere lengths at set joint angles compared to normal muscle from healthy subjects (22, 23, 35, 36). This sarcomere overstretch indicates that CP-affected muscles have fewer than normal sarcomeres in series, or reduced functional muscle length (27). However, these observations used typical developing muscles from separate subjects as controls rather than contralateral, noncontractured muscles in unilaterally affected subjects as was done in this study. This distinction is important given the variability in sarcomere lengths between subjects (37). In BPBI, experiments in animal models have indeed shown that contractures result from denervation-induced impairment of postnatal longitudinal muscle growth, independent of muscle strength imbalance (24, 25). Furthermore, the growth impairment is characterized by similar sarcomere overstretch as seen in human CP muscles, suggesting a potential common developmental etiology. The current study's findings confirm this common muscle length phenotype in both CP and BPBI through a direct comparison in humans, supporting a similar contribution of impaired longitudinal muscle growth to contractures in both conditions.

Simultaneously, the similarly reduced muscle strength in CP and BPBI in the current study argues against previously postulated mechanical etiologies of contracture pathogenesis involving imbalanced or excess muscle strength. In CP, muscles responsible for contractures often display spasticity due to disinhibition of the stretch reflex (38), leading to chronic abnormal dynamic joint posturing. For instance, the elbow typically assumes a flexed posture due to dysregulated elbow flexor muscle tone (39). Not surprisingly, the elbow subsequently develops a fixed elbow flexion contracture, wherein the elbow cannot achieve full passive extension even when the elbow flexor muscles are relaxed (6). However, treatments that reduce muscle strength and dynamic posturing in limbs affected by CP do not correct the associated contractures (20), calling into question the relationship between unopposed muscle strength and contracture development. Indeed, the abnormal muscles in CP have been found, as in the current study, to be functionally weaker than normal (40–43). Similarly, in BPBI, relative muscle overactivity has been thought to underlie contracture pathogenesis, since not all muscles are equally paralyzed by the brachial plexus injury, leading to imbalanced muscle activity across certain joints. However, imbalanced muscle strength cannot explain paradoxical contractures that occur in BPBI, including the common elbow flexion contracture (44–47). In typical cases of BPBI, the elbow flexor muscles (biceps brachii and brachialis) are denervated and flaccid while the triceps muscle that extends the elbow remains innervated and functional. As a result, the elbow initially assumes an extended posture, opposite to that seen in CP. However, an elbow flexion contracture ultimately develops (44–47), similar to that seen in CP, despite the opposite initial joint posturing. Indeed, elbow flexor strength has been found to be weaker than normal in subjects with BPBI-induced elbow flexion contractures (31), similar to that seen in the current study. Thus sufficient prior evidence exists to question the role of chronic joint posturing from unopposed or excessive muscle activity in contracture formation in BPBI as well as CP. The current study, by directly comparing both conditions, indeed demonstrates that contractures in both conditions are characterized by reduced rather than excessive muscle strength, together with similar deficits in muscle length despite opposite directions of dynamic joint posturing. These findings demonstrate that contractures in both conditions are a problem of reduced muscle length rather than excess muscle strength, suggesting that contracture treatments should be aimed at increasing muscle length rather than reducing muscle strength.

The characterization of muscle strength and length in the current study must be considered within the context of the methodologies used. Regarding muscle strength, isokinetic strength testing has been used in the lower extremity in CP (48–50), and in the upper extremity in children with BPBI (31) and CP (43), even in the setting of spasticity and co-contraction (43). Thus isokinetic strength testing is ideally suited for measurement of both muscle strength and functional muscle length in CP- and BPBI-induced elbow flexion contractures. However, the use of isokinetic strength testing in this study is not without limitations. While hemiplegic CP and unilateral BPBI offer a supposedly unaffected contralateral upper limb as a control, it could be argued that these limbs are not normal. Indeed, subtle contralateral upper limb motor deficits have been noted in unilateral BPBI (51) and hemiplegic CP (52). However, in these studies, strength, while altered in the unaffected arms, has been found to be reduced rather than increased compared to age appropriate norms, suggesting that the strength deficits noted in the affected limbs in our subjects is not the artifact of increased contralateral limb strength, such as might occur from overuse. In addition, none of the included subjects had deficits in passive elbow extension on the unaffected side compared to normative range of motion data (53), making such limbs suitable as non-contracture controls. Furthermore, the use of isokinetic dynamometry offers only indirect measures of muscle length, including the angle at which peak torque occurs and total workachieved. The highly variable shapes of the torque-angle curves made precise idendification of peak torque angle unreliable across subjects. However, impulse (area under the torque angle curve, also referred to as total work) was reliably measured, revealing significantly reduced impulse or total work in the affected elbows of both CP and BPBI subjects. Reduced total work correlates with reduced fascicle length in human testing and computational models (54) and with fewer sarcomeres in series in rat models (55). Therefore, the reduced total work seen in both CP and BPBI subjects, especially out of proportion to reductions in peak torque, suggests functionally shortened muscles. Furthermore, reduced peak torque correlates with reduced fascicle length as well (54), so it is conceivable that the reductions in peak torque can also be explained in part by reduced functional muscle lengths, rather than only weakness from abnormal innervation.

The sarcomere length measurement used in the current study remains an indirect measure of the number of sarcomeres in series in the muscle, which is the true measure of a muscle's functional length. However, the use of sarcomere overstretch to indicate too few sarcomeres in series has been well established (27). Moreover, the minimally invasive microendoscopy used to image sarcomeres *in situ* in the current study has been well validated (28, 29). In fact, this technology has recently been utilized to measure sarcomere lengths in human biceps brachii muscles in the setting of elbow flexion contracture following adult stroke (56). These investigators found sarcomere overstretch similar to that seen in our study, suggesting that the reduced functional muscle length characterizing contractures in CP and BPBI may be present in an even broader array of neuromuscular contractures.

An important limitation of the current study, as with all prior studies on CP- and BPBI-induced contractures in human subjects, is that it assesses the contracture phenotype only after the contractures have formed. Thus cause and effect cannot be ascertained. Instead, only experimental manipulation in animal models during contracture formation can be used to elucidate causative mechanisms. Such investigations in animal models of BPBI have recently demonstrated that impaired longitudinal muscle growth and contractures arise from increased protein degradation by the ubiquitin-proteasome pathway (57). Importantly, in these animal models, pharmacologic inhibition of the proteasome has been found to prevent contractures and rescue sarcomere overstretch (57, 58), representing a novel approach to prevent contractures by targeting an underlying biological mechanism. While this precise approach is not immediately translatable to humans given the toxicity of proteasome inhibitors, the use of such animals models for pre-clinical discovery and testing of contracture prevention and treatment strategies for BPBI is supported by the current study demonstrating relevance of the animal contracture phenotype to the human phenotype. Moreover, the similarity identified by the current study in muscle contracture phenotype between BPBI and CP supports potential translation of such approaches to CP as well, which is especially important in light of the lack of an animal model of CP-induced contractures (59). How BPBI as a peripheral nerve injury and CP as a brain problem could lead to a final common pathway of muscle contracture remains to be elucidated, although evidence that BPBI affects brain development (60) suggests that the two conditions may share additional overlapping perturbations of neuromuscular development.

Overall, the current study identifies similarties in muscle contracture phenotype between CP and BPBI that challenge the presumed dichotomy between upper and lower motor neuron lesions in contracture pathogenesis, potentially allowing translation of research findings across conditions. Most importantly, however, this study reveals the broader concept of “myobrevopathy”, or disorder of short muscle, as a common phenotypic feature spanning otherwise disparate neuromuscular conditions. This final implication argues for additional research into specific mechanisms regulating muscle length, a long-overlooked dimension in muscle biology.

## Data Availability

The original contributions presented in the study are included in the article/Supplementary Material, further inquiries can be directed to the corresponding author/s.
